# Periodic limb movements in Parkinson’s disease: a critical review of pathophysiology and a framework for clinical management

**DOI:** 10.1038/s41531-026-01356-1

**Published:** 2026-04-15

**Authors:** Shihao Yi, Hanshu Liu, Xinyu Hu, Jiaming Xu, Shurui Zhang, Huaqing Zhang, Zhixin Wang, Tianqi Han, Liang Kou, Yun Xia, Tao Wang, Zhicheng Lin, Nian Xiong

**Affiliations:** 1 Department of Neurology, Union Hospital, Tongji Medical College, Huazhong University of Science and Technology, Wuhan, Hubei Province China; 2 Laboratory of Psychiatric Neurogenomics, McLean Hospital; Harvard Medical School, Belmont, MA USA

**Keywords:** Diseases, Neurology, Neuroscience

## Abstract

Periodic limb movements (PLM) refer to periodic episodes of repetitive and stereotypical limb movements, predominantly affecting the lower extremities. The prevalence of PLM is estimated at 17.6% to 86.7% in patients with Parkinson’s disease (PD), which is much higher than 4% to 11% in the general population. PD is a chronic neurodegenerative movement disorder characterized by both motor and non-motor symptoms (NMS), and PLM represents one of the common non-motor manifestations. PLM in PD has garnered increasing attention due to its high prevalence, association with reduced sleep quality, elevated risk of comorbidities, unclear pathophysiology, and limited treatment options. This review outlines the epidemiology and risk factors for PLM in PD patients. We explore several underlying mechanisms including iron deficiency, dopaminergic dysregulation, and sympathetic nervous activity. Treatment strategies for PLM comorbid with PD are broadly categorized into non-pharmacological and pharmacological therapies where dopamine agonists can alleviate symptoms of both PD and PLM. Clarifying the relationship between PLM and PD is essential, as it may lead to expanded novel treatment approaches and improved quality of life for affected patients.

## Introduction

Parkinson’s disease (PD) is a progressive and latent neurodegenerative movement disorder characterized neuropathologically by the accumulation of abundant α-synuclein aggregates in the form of Lewy bodies and degeneration of dopaminergic neurons in the substantia nigra pars compacta^1–3^. With PD traditionally defined by its cardinal motor symptoms including tremor, rigidity, bradykinesia, and instability, growing clinical evidence highlights the significance of the non-motor symptoms (NMS)^4,5^. These prodromal features, comprising sleep disorders, depression, anxiety, executive dysfunction, and sensory abnormalities, frequently emerge years to decades prior to the onset of classic motor symptoms, and have drawn increasing attention in that these NMS may serve as early identification of pre-PD^6^.

As one of the most significant NMS in PD, sleep disorders, including insomnia, excessive daytime sleepiness, circadian dysfunction, obstructive sleep apnea (OSA), rapid eye movement sleep behavior disorder (RBD), restless legs syndrome (RLS), and periodic limb movements (PLM), have affected approximately 80% of PD patients after 5 years of diagnosis^7,8^. PLM has emerged as a key area of interest, drawing increasing research attention regarding morbidity, clinical manifestations, pathophysiological mechanisms, diagnostic criteria, treatment, and comorbidity with PD^9,10^.

PLM refers to repetitive, stereotyped, and involuntary limb contractions primarily affecting lower extremities that may occur either during wakefulness or in sleep^11,12^, and the objective diagnosis of PLM is primarily based on polysomnography (PSG). While PLM serves as a clinical symptom, periodic limb movement disorder (PLMD) constitutes a diagnosis of exclusion. PLMD requires PSG-confirmed periodic limb movement during sleep (PLMS) at a frequency of ≥5 events/hour in children or ≥15 events/hour in adults, accompanied by clinically significant sleep disturbance or daytime symptoms that are not better explained by another disorder^13^. It has been reported that the prevalence of PLM ranges from 5 to 8% in children and 3.9 to 11% in healthy adults^14^. Furthermore, a study has indicated that a high prevalence of PLM occurs in PD patients^15^. Prior research has shown that the prevalence of PLM in RBD patients who eventually develop PD is 82%, with a significantly higher periodic limb movement index (PLMI) compared to those who did not progress to PD^16^. Research also has indicated that PD patients frequently exhibit PLM, with those demonstrating a PLMI ≥ 15 displaying more severe PD symptoms compared to those with a PLMI < 15^17^. In addition, a clinical trial demonstrated that treatment with cabergoline, a dopamine D2 receptor agonist, significantly reduced PLM in PD patients, highlighting the role of dopaminergic dysfunction in the pathophysiology of PLM-PD comorbidity^18^. PLM are associated with conditions such as RLS^19^, cognitive impairment^20^, and precapillary pulmonary hypertension^21^, suggesting shared pathophysiological mechanisms or systemic consequences of sleep-related motor dysregulation. Accordingly, such comorbidities warrant a mechanistic understanding. In this review, we comprehensively review the epidemiology, risk factors, pathophysiology mechanism, diagnostic criteria, and treatments of PLM in PD.

## Epidemiology

Numerous studies from 2003 to 2022 have indicated a significant incidence of PLM in PD patients across different countries (Table 1).

**Table 1:** The prevalence of PLM in PD patients

Study (Country)	Study Type	Sample Size	Measures for PLM	Mean Age (Years Old)	Male Proportion	Disease Duration (Years)	Anti-PD Medications (Mean LEDD)	Prevalence
Happe S et al., 2003 (Austria)^22^	Observational study	11	PSG (PLMI ≥ 5)	65.8	63.6%	5.8	209.1 mg	45%
Lavault S et al., 2009 (France)^25^	Case-Control study	24	PSG (PLMI ≥ 15)	70.0	75.0%	9.5	807 mg	29.5%
Norlinah MI et al., 2009 (Malaysian)^23^	Cross-sectional study	44	PSG (PLMI ≥ 5)	64.0	59.0%	5.8	PLM: 150 mg No PLM: 300 mg	32%
Covassin N et al., 2012 (USA)^17^	Observational study	45	PSG (PLMI ≥ 15)	68.5	71.0%	5.8	462.71 mg (PLMI < 15) 628.85 mg (PLMI ≥ 15)	57.8%
Chahine LM et al., 2013 (USA)^26^	Cross-sectional study	62	PSG (PLMI ≥ 15)	63.9 (median)	66.1%	5 (median)	502.5 mg (median)	21.0%
Puligheddu M et al., 2014 (Italy)^30^	Prospective study	44	PSG (PLMI ≥ 15)	67.6	63.6%	3.2	427.8 mg	25.0%
Prudon B et al., 2014 (UK)^29^	Prospective study	106	Actigraphy (PLMI ≥ 5)	66.5 (median)	63.2%	4.8 (median)	140 mg (median)	49.5%
Martinez-Ramirez D et al., 2015 (Mexico)^28^	Cross-sectional study	55	PSG (PLMI ≥ 15)	61.9	61.8%	6.5	635.4 mg	24.5%
Scullin MK et al., 2015 (USA)^27^	Cross-sectional study	34	PSG (PLMI ≥ 15)	62.4	62.0%	6.8	531.3 mg	About 50.0%
Jasti DB et al., 2018 (India)^31^	Observational study	60	PSG (PLMI ≥ 5)	60.2	76.7%	4.4	Not mentioned	86.7%
Shen Y et al., 2020 (China)^32^	Retrospective study	239	PSG (PLMI ≥ 15)	64.2	65.7%	4.0	250.0 mg	17.6%
Hermann W et al., 2020 (Germany)^12^	Cross-sectional study	44	PSG (PLMI ≥ 15)	68.9	57%	3.8	453.0 mg	39%
Sun S et al., 2021 (China)^33^	Observational study	81	PSG (PLMI ≥ 15)	62.3	44.4%	3.7	317.2 mg	33.3%
Tsuru A et al., 2022 (Japan)^34^	Retrospective study	118	PSG (PLMI ≥ 15)	67.1	61.0%	6.7	491.2 mg	22.9%
Jiang Y et al., 2023 (China)^35^	Cross-sectional study	44	PSG (PLMI ≥ 15)	68.9	68.2%	1.3	All untreated	27.3%

*LEDD* levodopa equivalent dose daily; *PLM* periodic limb movement; *PLMI* periodic limb movement index; *PSG* polysomnography.

In 2003, Happe S et al. quantified the frequency of PLMS occurrence in idiopathic PD patients from Austria using PSG and reported a PLMI > 5 in 45% of PD patients^22^. In 2009, a cross-sectional study involving 44 Malaysian PD patients found that 32% exhibited PLM of varying severity: 42.8% mild, 28.6% moderate, and 28.6% severe^23^. This classification adhered to the International Classification of Sleep Disorders, 2nd edition standard, which defined PLM severity thresholds as mild (PLMI 5-25 events/hour), moderate (PLMI 25.1–50 events/hour), and severe (PLMI > 50 events/hour)^24^. During the same period, a French case-control study reported that 29.5% of PD patients had a PLMI > 15, which was 3.7-fold higher than the 8% prevalence of PLM observed in controls^25^. Between 2012 and 2015, several cross-sectional and observational studies among U.S. PD patient populations showed substantially higher prevalence rates of PLM, with multi-center research estimating prevalence rates ranging from 21% to 57.8%^17,26,27^. Moreover, a study conducted in Mexico reported that 24.5% of PD patients satisfied the diagnostic criteria for PLMD, and further demonstrated that a lower levodopa equivalent dose was associated with PLMD through univariate and multiple logistic regression analyses^28^. In addition, two prospective studies from European populations reported the prevalence of PLM ranging from 25% to 49.5% in PD patients^29,30^. In 2018, Jasti DB et al. evaluated sleep quality and disorders in 60 PD patients from rural areas in India and found that 86.7% of those patients exhibited PLMD (defined as PLMI > 5) by various relevant scales and PSG with moderate (25.1 < PLMI < 50) and severe (PLMI > 50) present in 23.3% and 16.7% of patients, respectively. Notably, their study also reported a significant correlation between PD disease severity and PLMI (*P* = 0.023) / PLM with arousal (*P* = 0.012), indicating advanced PD stages predict more severe and obvious PLMD symptoms^31^.

Recently published in 2020, a retrospective study from China utilizing PSG with a PLMI threshold ≥15 reported a PLM prevalence of 17.6% in PD patients^32^. Applying similar diagnostic criteria, a subsequent observational study from China in 2021 found a higher prevalence rate of 33.3%^33^. During the same period, a cross-sectional cohort study was the first to investigate the association between motor symptoms asymmetry and PLMS in PD. Their results revealed that patients with PD, sleep disorder breathing (SDB), and RLS all exhibited significant side-to-side differences in PLM, PLMI, and PLM with arousal index. Furthermore, only 39% of PD patients had a PLMI greater than 15, which was significantly lower than the rates observed in SDB (80%) and RLS (88%) patients^12^. A 2022 retrospective study involving 118 PD patients in Japan with varying disease severity found that 22.9% were diagnosed with PLMD^34^. In 2023, a cross-sectional study from China included 44 drug-naïve PD patients and explored the impact of PD on sleep structure, finding that 27.3% of those patients had PLMD. This study subsequently demonstrated that PLMI increased with higher NMS questionnaire scores in poor sleepers, indicating that untreated PD patients with sleep disturbances had higher PLMI corresponding to more severe NMS^35^.

The variation in PLM prevalence among PD patients across different countries may be attributed to several variables, including differences in sample size, individual variation in disease severity, differential use of anti-PD medications, and inconsistent diagnostic criteria. Therefore, large-sample and multi-center studies with stringent inclusion/exclusion standards are required in the future to accurately estimate the prevalence of PLM in PD patients.

## Risk factors

Recent research has demonstrated that several risk factors may increase the prevalence of PLM in PD patients, including gender, age, inflammation, *Helicobacter pylori* (HP), and genetics^17,36^.

Current research has not clearly established whether gender and age are risk factors for PLM in PD patients. However, we speculate that they may be potential risk factors based on studies of PLM in the general population. A genome-wide association study of 6843 subjects across four cohorts revealed that the PLMI increased by 0.49/hour annually, confirming age as an independent risk factor for PLM^37^. Notably, the relationship between age and PLM was particularly evident in patients with PLMI ≥ 15^38^. Similarly, a sleep cohort study involving 2356 white males revealed that the mean age of participants with a PLMI > 15 was significantly higher than that of those with a PLMI < 15, indicating that advanced age may be associated with more severe and frequent PLM symptoms^39^.

Although age is the greatest risk factor for PD, gender also contributes to the higher incidence, prevalence, and mortality of PD observed in males compared to females^40,41^. Similarly, a SHIP-TREND cohort from German population of 1107 subjects reported that the male gender was identified as a significant independent risk factor for PLMI > 15^42^. Moreover, Haba-Rubio J et al. reported consistent results with males exhibiting a rate of PLMI > 15 that is 1.56 times higher than that of females, further confirming male gender as a risk factor for PLM^43^. While gender and age are established risk factors for PLM or PD, current studies have not clearly established the relevance of these elements in PD patients with PLM, and longitudinal studies with large samples are essential to investigate this relationship.

Neuroinflammation plays a vital role in the development and progression of PD. Multiple studies of peripheral blood and cerebrospinal fluid from PD patients have revealed factors associated with inflammation are able to exacerbate neuroinflammation and further promote neurodegenerative progression^44^. A cross-sectional study showed significantly elevated levels of inflammatory markers, such as lipoprotein-associated phospholipase A2 and highly sensitive-C-reactive protein (CRP), in the PLMI > 15 group compared to PLMI < 15 group^45^. Similarly, plasma levels of CRP and fibrinogen were significantly higher in OSA patients with comorbid PLMS than in those with OSA only; multivariate analysis further revealed that PLMS were independently associated with elevated plasma CRP and fibrinogen levels^46^. Thus, PLM may contribute to pro-inflammatory microenvironment that aggravates pathological protein aggregation and promotes neurodegeneration, elevating PD susceptibility^47^.

Multiple studies have indicated a close association between HP infection and PD, suggesting that HP can impair levodopa efficacy and exacerbate motor symptoms in PD patients^48,49^. Furthermore, a 2020 meta-analysis found that HP infection increased the risk of PD through potential mechanisms involving the promotion of 1-methyl-4-phenyl-1,2,3,6-tetrahydropyridine synthesis and inflammation activation, both of which exacerbate PD symptoms^50^. Iron deficiency, one of the mechanisms contributing to PLM, has been confirmed in patients with HP infection^51^. A recent large-scale study involving 9393 HP-infected patients and 37,572 uninfected controls revealed a significant association between HP infection and PLMD, pointing to a higher risk of PLMD in infected individuals. This risk was particularly elevated in males, patients aged ≥65 years, and those with infection durations exceeding five years^52^. Collectively, these studies suggest that PD patients with comorbid HP infection may have a high prevalence of PLM. However, current research specifically investigating the impact of HP eradication on PLM symptoms in patients with PD remains limited, and further clinical studies are required to validate this potential therapeutic strategy.

Unlike *SNCA* or *LRRK2*, the *BTBD9* variant rs3923809 associated with PLM has not been consistently identified as a risk factor for PD itself, suggesting that PLM in PD may arise from a complex interaction between PD-specific neurodegeneration and the patient’s underlying genetic susceptibility to sleep movement disorders. However, it is necessary to note that the current understanding of these susceptibility genes is predominantly extrapolated from large-scale studies in the general population rather than specific PD cohorts.

For instance, a prior study in a non-PD population identified rs3923809 as a primary genetic determinant of PLMS, demonstrating that the A allele of rs3923809 is significantly associated with increased PLMS severity. The presence of each A allele correlated with a 5.5% increase in the ferritin index and a 13% decrease in serum ferritin, indicating impaired iron metabolism contributes to PLMS pathophysiology. This study further identified three candidate susceptibility genes for PLMS, including *BTBD9*, *GLO1*, and *DNAH8*^53^. Similarly, a genetic association study of 1086 participants from the Wisconsin Sleep Cohort identified significant correlations between PLM and 13 single nucleotide polymorphisms (SNPs) in 6 loci previously linked to RLS susceptibility: *BTBD9*, *TOX3/BC034767*, *MEIS1*, *MAP2K5/SKOR1*, and *PTPRD*. Notably, *BTBD9* exhibited the strongest association with PLM, whereas *MEIS1* had a comparatively greater impact on RLS^54^. Additionally, the Osteoporotic Fractures in Men Study assessed the association between SNPs and PLMS in 2356 white men, identifying significant associations of SNPs within the *BTBD9*, *MEIS1*, and *MAP2K5/SKOR1* genes with a PLMI > 15^39^. Building on previous research, a recent genome-wide association study of PLMS across four cohorts including the Osteoporotic Fractures in Men Study, the Wisconsin Sleep Cohort Study, HypnoLaus, and the Multi-Ethnic Study of Atherosclerosis confirmed significant genetic associations between PLMS and the *BTBD9* and *MEIS1* loci. Furthermore, linkage disequilibrium score regression analysis demonstrated that PLMI was significantly genetically correlated with RLS, insomnia, and stroke^37^.

Despite these robust findings in the general population, there is currently a paucity of research specifically investigating these genetic risk factors within PD patients. Whether these variants carry the same risk magnitude or interact differently within the neurodegenerative context of PD remains to be elucidated by future PD-oriented genetic studies.

## Clinical manifestations of PLM in PD patients

In the general population, PLM is characterized by periodic, repetitive, and stereotypical limb movements with the lower extremities being particularly susceptible^55^. However, PD patients with comorbid PLM exhibit more severe motor, and NMS beyond these characteristic manifestations^17^.

Emerging evidence indicates PD patients with a high burden of PLM may exhibit more severe motor symptoms. While a recent meta-analysis confirmed that PD patients generally present with higher PLMI values compared with healthy controls^9^, specific correlations with disease severity have also been identified. For instance, a comparative study revealed significant correlations between PLMI and Unified Parkinson’s Disease Rating Scale (UPDRS) scores (Total, Part II, and Part III), suggesting that elevated PLMI parallels worsening motor dysfunction^17^. Furthermore, disease progression, as measured by the Hoehn & Yahr scale, has been shown to correlate significantly with PLM scores^56^, indicating advanced disease stages may be predictive of more frequent PLM symptoms accompanied by severe motor impairment.

Asymmetric PLM distribution was present in populations with PD, SDB, and RLS; however, this PLM pattern occurred less frequently in PD patients than in RLS or SDB and exhibited greater nocturnal stability. Critically, no association was found between asymmetric PLM distribution and motor symptoms asymmetry in PD^12^. PLM occurs not only during sleep in PD patients but also manifests during wakefulness. A 2022 case series reported periodic, stereotyped lower-extremity movements during wakefulness in four PD patients with a history of PLMS. These limb movements, termed periodic limb movement during wakefulness (PLMW), phenomenologically resemble PLMS but typically emerge during the daytime. Moreover, PLMW occurrence was temporally associated with levodopa wearing-off periods and demonstrated significant responsiveness to dopaminergic therapy. Consequently, the study characterized PLMW as a wearing-off phenomenon in PD, classifying it within the spectrum of low-dose.

Compared with motor symptoms, PLM demonstrates a stronger impact on NMS by contributing to various conditions such as sleep disturbance, RBD, RLS, cognitive dysfunction, and psychiatric diseases in PD patients. Specifically, among PD patients with poor sleep quality, PLMI showed a significant positive association with Non-Motor Symptoms Questionnaire scores. Linear regression analysis further revealed that PLMI explained 31.6% of the Non-Motor Symptoms Questionnaire score variation, indicating that higher PLMI levels contribute to greater NMS severity and may serve as an independent predictor of NMS aggravation^35^.

Regarding sleep architecture and RBD, PD patients with a higher PLMI exhibit sleep abnormalities, including reductions in total sleep time (TST), sleep efficiency (SE), N2 sleep, slow wave sleep (SWS), and rapid eye movement (REM) sleep, along with increases in N1 sleep, wake time after sleep onset, rapid eye movement latency, and apnea hypopnea index^9^. Notably, PLM burden is closely linked to RBD comorbidity. A recent study reported that the prevalence of PLMI ≥ 15 in PD patients comorbid with RBD was significantly higher than in those without this comorbidity^57^, underscoring the increased risk of concurrent RBD in patients with elevated PLM burden. Furthermore, lower scores on the mini-mental state examination, which assesses the severity of cognitive impairment, were significantly associated with reduced TST and SWS, indicating that PD patients with more severe PLM symptoms are likely to experience cognitive dysfunction as a concomitant condition of their sleep disturbance^9^.

Similarly, a study analyzing demographic, clinical, and polysomnographic data compared PD patients with and without RLS. The prevalence of PLM was higher in the PD with RLS group (37%) than in the PD without RLS group (31.5%). Results further demonstrated that the PLMI was negatively correlated with TST and SE but positively with sleep latency in the PD with RLS group. Additionally, multiple linear regression analysis revealed that PLMI was the most significant predictor of reduced SE, exceeding the impact of other risk factors such as PD duration and UPDRS III scores^33^. The NMS of PD includes psychiatric symptoms such as hallucinations, as well as sleep disorders^58^. A single-center retrospective cross-sectional study revealed significantly higher PLMI values in PD patients with hallucinations compared to those without. These patients also exhibited poor sleep quality, cognitive impairment, anxiety, and depression alongside their hallucinations^59^.

## The pathophysiology of PLM in PD

**Fig. 1:**
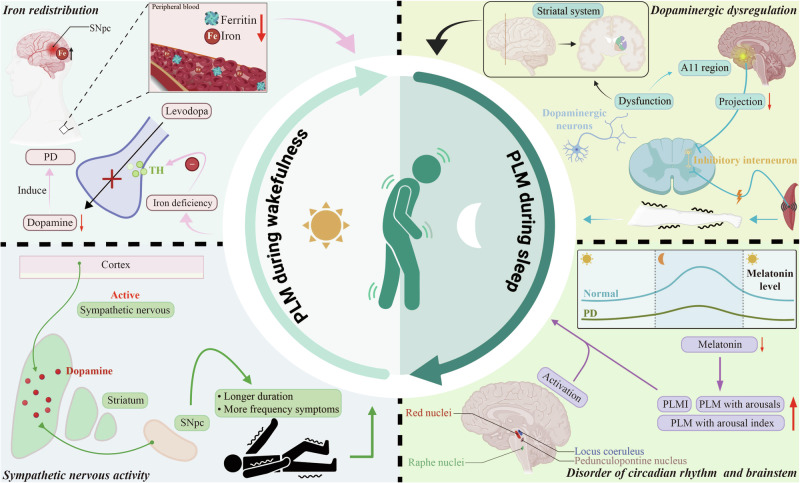
Potential pathophysiological mechanisms of PD with comorbid PLM. PLM may occur during both wakefulness and sleep, and are closely associated with PD. Iron deficiency impairs the conversion of levodopa to dopamine via TH, which contributes to dopaminergic dysfunction. Moreover, the risk of developing PLM in PD populations is increased through several mechanisms, including iron redistribution, dopaminergic dysregulation, sympathetic nervous activity, and disturbances of circadian rhythm and brainstem function.Abbreviations:PD, Parkinson’s disease; PLM, periodic limb movement; PLMI, periodic limb movement index; SNpc, substantia nigra pars compacta; TH, tyrosine hydroxylase.

PLM occurs in approximately 90% of RLS patients^60^. Currently, most research on PLM mechanisms is based on models or patients with RLS-associated PLM^11^, and direct mechanistic evidence specific to PD remains limited. Therefore, the possible pathophysiology of PLM in PD is largely extrapolated from RLS and general population data, which is reviewed through four primary aspects: iron deficiency, dopaminergic dysregulation, sympathetic nervous activity, and other potential mechanisms, primarily using RLS-associated PLM models (Fig. 1).

Iron deficiency has been demonstrated as one of the pathophysiological mechanisms in RLS/PLM^61,62^. A cross-sectional study involving 899 individuals revealed that patients with PLM and a PLMI > 15 exhibited significantly lower serum ferritin levels (<50 ng/ml)^63^. Since iron acts as an essential cofactor for tyrosine hydroxylase (TH), which is the rate-limiting enzyme for dopamine synthesis, iron deficiency directly inhibits TH activity, impairing dopaminergic signaling and decreasing dopamine production^64,65^. In addition, iron in mitochondria is stored as ferritin and used for the biosynthesis of heme and iron-sulfur clusters, which facilitate oxidation-reduction reactions and participate in the electron transport chain^66^. Therefore, insufficient iron impairs synthesis of heme and iron-sulfur clusters, resulting in oxidative stress and an increase in reactive oxygen species production, which eventually aggravates the progression of PD^67,68^. A recent study has also indicated that iron deficiency can activate microglia via iron regulatory protein 1 and increase the release of inflammatory cytokines (tumor necrosis factor-alpha (TNF-α) and interleukin-1 beta (IL-1β)), causing dopaminergic neuronal degeneration and DNA damage^69^.

Although iron overload is well-documented in PD patients with significant accumulation observed in the substantia nigra pars compacta (SNpc)^70^, evidence regarding systemic iron levels has evolved. While earlier studies reported no significant difference in serum, plasma, and cerebrospinal fluid iron levels between PD patients and controls^71^, a recent meta-analysis has indicated that serum iron levels are significantly lower in PD patients, despite a trend towards elevated cerebrospinal fluid iron levels^72^. A prior study further demonstrated that total iron levels increased by 31%-35% in the SNpc but decreased by 29% in the globus pallidus of PD patients, while no significant changes were observed in other brain regions^73^. These selective alterations in PD patients’ iron content were corroborated by multiple techniques including histochemistry and biochemical methods^74^. Additionally, decreased levels of iron, ferritin, and total iron-binding capacity have also been reported in late-stage PD patients^75^. This apparent contradiction observed in PD patients may arise from altered iron distribution, specifically aggregation within the SNpc despite unchanged or reduced peripheral blood iron concentrations^76^.

In brief, when iron deficiency in patients with PLM reaches a critical threshold, it reduces dopamine synthesis while exacerbating oxidative stress and activating inflammatory responses. These synergistic effects may promote dopaminergic neuronal damage, thereby contributing to the development of PD. Conversely, the altered iron distribution observed in PD, characterized by accumulation of specific central regions despite reduced peripheral levels, is hypothesized to provide a pathological basis for the onset of PLM. Although studies specifically investigating iron indices in PD patients comorbid with PLM are currently limited, this pattern suggests a pathology of aberrant redistribution, which may serve as a risk factor in the concurrent development of PD and PLM.

Evidence from PLM patients, RLS patients and mouse models indicates that dopaminergic dysfunction and circadian rhythm dysregulation are the primary mechanisms underlying PLM^77–79^. Anatomical evidence from RLS patients indicates that dysfunction of dopaminergic neurons in the A11 region of the dorsal-posterior hypothalamus reduces spinal projections, thereby decreasing inhibitory interneuron activity in the spinal dorsal horn and disinhibiting signaling from high-threshold muscle afferents^78^. This is supported by rat models with 6-hydroxydopamine-induced lesions of the A11 nuclei, which exhibit significantly elevated wakefulness and limb movements that are reversed by acute dopamine agonist treatment, mirroring the clinical presentation of human PLM^80^. Corroborating these findings, postmortem studies have demonstrated that PD is associated with selective dopaminergic degeneration in the A11 region^81,82^. In addition to this structural deficit, physiological dopamine levels follow a circadian rhythm characterized by a nocturnal nadir, particularly during non-rapid eye movement (NREM) sleep^83^. In PD patients, this nocturnal decline exacerbates the pre-existing depletion, further compromising spinal inhibition and facilitating PLM. Moreover, PLM generally occurs at night and is primarily concentrated in the N2 stage of NREM sleep rather than REM sleep^84,85^. This interaction elucidates the nocturnal predominance of PLM in PD. Thus, the synergistic effect of A11 neurodegeneration and the nocturnal dopamine nadir disrupts the hypothalamic A11-spinal pathway, potentially predisposing PD patients to excessive lower limb movements during NREM sleep and manifesting as PLM.

Along with hypothalamic dopaminergic neurons, the striatal dopaminergic system significantly influences PD symptoms^86^. A previous study demonstrated that PD patients with a higher PLMI exhibited lower striatal dopaminergic transporter binding, indicating that the degree of striatal dopaminergic degeneration was significantly associated with PLMI, as assessed by PSG and β-CIT single photon emission computed tomography^22^. In addition, a 6-[^18^F]fluoro-L-dopa positron emission tomography study revealed mild nigrostriatal presynaptic dopaminergic hypofunction in patients with PLMD and RLS^87^. Striatal dopamine maintains normal wakefulness, and its depletion causes fragmentation of the sleep-wake cycle and leads to abnormal sleep behaviors^88,89^. Collectively, the concurrent dysfunction of the hypothalamic and striatal dopaminergic systems may constitute the core mechanism underlying the exacerbated severity of PLM symptoms and the occurrence of associated sleep disorders in patients with PD.

A close association has been demonstrated between increased sympathetic nervous activity and PD/PLM^90,91^. Wu et al. reported that patients with elevated PLMI exhibit heightened sympathetic nervous activity in autonomic regulation compared to those without PLMS^92^. Accordingly, strong sympathetic activation, which facilitates extrapyramidal motor networks and contributes to PLM, is significantly associated with higher PLMI and longer movement duration^93^. Since PD is fundamentally characterized by degeneration of the nigrostriatal dopaminergic system and compromised motor regulation^94^, it is plausible that PLM in PD arises from a pathogenic interplay between sympathetic overdrive and extrapyramidal impairment, where autonomic hyperactivity exacerbates motor disinhibition.

Complementary to the primary dopaminergic mechanisms, circadian rhythm disruption and brainstem neurodegeneration contribute to the pathophysiology of PLM in PD. PD is characterized by circadian misalignment and blunted nocturnal melatonin secretion^95^. Animal and human studies indicate that this disruption correlates with sleep-wake disturbances^96^. Physiologically, melatonin exerts a suppressive effect on nocturnal motor activity; its depletion therefore leads to disinhibition. Melatonin treatment has been shown to effectively improve non-motor symptoms and sleep quality in PD patients^97^. Moreover, a clinical trial confirmed that melatonin significantly reduced PLM-associated parameters, including PLMI, PLM with arousals, and PLM with arousal index^98^. Thus, circadian rhythm dysfunction may constitute a key pathogenic factor for PLM, and exogenous melatonin mitigates these symptoms by enhancing the physiological circadian inhibition of locomotor activity.

Moreover, brainstem neurodegeneration in PD disrupts physiological sleep architecture and compromises the descending inhibitory control over spinal motor pathways^99^. Neuropathological evidence from a study on PD indicated that sleep disturbances are significantly linked to α-synuclein accumulation in the locus coeruleus and raphe nuclei^100^. Numerous studies have shown that lesions and neurodegeneration in the brainstem including the locus coeruleus, raphe nuclei, and pedunculopontine nucleus, have a significant impact on NMS in PD, particularly sleep disorders^101,102^. Dysfunction in this system reduces the threshold for motor activation during sleep^77^. A case report described a patient who developed PLM, RLS, and altered sleep architecture following an ischemic infarction in the right lenticulostriate region, demonstrating the necessity of the basal ganglia-brainstem axis for motor suppression^103^. Similarly, high-resolution functional MRI in patients with RLS/PLM revealed direct activation of the red nuclei and brainstem during movement episodes^104^. Thus, extrapolating from PLM/RLS and general PD population cohorts, brainstem abnormalities in PD are theorized to facilitate PLM by simultaneously destabilizing sleep architecture and disinhibiting downstream spinal motor neurons.

Currently, the primary pathogenesis of PD involves the pathological aggregation of α-synuclein, oxidative stress, ferroptosis, and neuroinflammation^105^, providing a potential pathophysiological basis for PD-PLM comorbidity. However, among the existing evidence, only the degeneration of dopaminergic neurons in the hypothalamic A11 region and the aberrant aggregation of α-synuclein in brainstem nuclei such as the locus coeruleus and raphe nuclei have been established as PD-specific mechanisms. In contrast, the exact roles of oxidative stress and neuroinflammation mediated by abnormal iron redistribution, as well as sympathetic overactivation and circadian disruption in this comorbidity remain to be further elucidated.

## The diagnostic criterion and differential diagnosis of PLM/PLMD in PD

Patients diagnosed with PD who also meet the diagnostic criteria for PLM/PLMD receive a diagnosis of comorbid PLM/PLMD with PD (Table 2). World Association of Sleep Medicine, the International Restless Legs Syndrome Study Group, and the European Restless Legs Syndrome Study Group first described candidate leg movements (CLM) as unilateral or bilateral leg movements lasting between 0.5 and 10 s, and then defined PLM as a series of at least four consecutive CLM documented by PSG, with inter-movement intervals ranging from 10 to 90 s^106^. In addition, the International Classification of Sleep Disorders-Third Edition defines PLMD as the presence of PLMS ( ≥ 5 events/hour in children or ≥15 events/hour in adults) measured by PSG with clinically significant sleep disturbance or daytime symptoms, where these symptoms are not better explained by another disorder^13^. The gold standard for diagnosing PLM/PLMD is PSG, and other diagnostic instruments such as actigraphy or Periodic Activity Monitor-Restless Legs (PAM-RL) device are also employed to detect these conditions^107,108^. Moreover, PLMD symptoms may closely resemble those of depression symptoms leading to incorrect treatment^109^. Consequently, it is essential to differentiate between PLMD and these conditions.

**Table 2:** Definitions and diagnostic criteria of PLM and related terms in PD

Term	Diagnostic criteria
PLM	1. Presence of CLM, defined as unilateral or bilateral leg movements lasting between 0.5–10 s. 2. At least four consecutive CLM documented by PSG. 3. Intervals between adjacent CLM ranging from 10 to 90 s.
PLMS	PLM occurs during sleep.
PLMW	PLM occurs during wakefulness.
PLMD	1. Presence of PLMS confirmed by PSG. 2. PLMI > 5/hour in children or >15/hour in adults. 3. Clinically significant sleep disturbance or daytime symptoms attributed to PLMS. 4. PLMS and related sleep symptoms are not better explained by other sleep disorders.

*CLM* candidate leg movements; *PLM* periodic limb movement; *PLMD* periodic limb movement disorder; *PLMI* periodic limb movement index; *PLMS* periodic limb movements during sleep; *PLMW* periodic limb movements during wakefulness; *PSG* polysomnography.

PLM is reported in various neurodegenerative conditions, including Alzheimer’s disease^110,111^ and atypical parkinsonism such as dementia with Lewy bodies^112–114^, multiple system atrophy^115,116^, progressive supranuclear palsy^117,118^, and corticobasal degeneration^119^. Notably, progressive supranuclear palsy is distinguished by markedly elevated PLMI values, which are significantly higher than those observed in PD patients^117,118^. In contrast, PLMD is identified through objective PSG evidence of repetitive limb movements and sleep disturbance in patients who are often unaware of the movements^120,121^. Furthermore, differentiating PLMD from RLS is essential for PD management. While RLS shares therapeutic similarities with PLMD^122,123^, it is distinctively a clinical diagnosis driven by a subjective circadian urge to move the legs that worsens at rest and is relieved by movement^124^. Consequently, PLMD serves as a diagnosis of exclusion in PD, confirmed only after RLS and other sleep disorders have been ruled out^125^.

Furthermore, diphasic dyskinesia, a subtype of levodopa-induced dyskinesia accounting for approximately 20% of cases, shares phenomenological similarities with PLMW^126^. Specifically, diphasic dyskinesia typically manifests as prominent stereotypical movements in a unilateral lower limb, occasionally accompanied by dystonia, coinciding with the onset of levodopa action. These movements subsequently spread to involve ipsilateral and contralateral limbs, subsiding during the peak-dose period, and re-emerging as levodopa levels decline^126,127^. In contrast, PLMW is distinguished by smaller movement amplitudes, the absence of dystonia, and strict periodicity, predominantly occurring during the wearing-off phase^128^. Collectively, these characteristics allow for clear clinical differentiation.

Beyond RLS and diphasic dyskinesia, differentiating PLM phenotypes between PD and idiopathic RLS carries important clinical implications. PLM in PD, compared with that in idiopathic RLS^129,130^, is more frequently associated with comorbid RBD and exhibits a higher PLMI during REM sleep^15,17^, yet demonstrates less asymmetric limb movement distribution and greater nocturnal stability^12^. In RLS, inter-limb movement intervals display a characteristic bimodal distribution, and the movements are typically accompanied by a subjective urge to move and sensory discomfort^131,132^, whereas PLM in PD is predominantly identified through objective monitoring and is frequently asymptomatic^29^. Notably, PLMS in PD has been associated with executive dysfunction, with higher PLMI values correlating with greater executive impairment^27^. Pathophysiologically, PLM in RLS is primarily driven by Central nervous system iron deficiency or peripheral iron metabolism abnormalities^133^. In contrast, PD-associated PLM is predominantly attributable to nigrostriatal dopaminergic neuronal degeneration^22,134^. From a therapeutic perspective, long-term dopaminergic treatment in RLS exhibits a significant risk of symptom augmentation^135^, while in PD the therapeutic response is principally influenced by the wearing-off phenomenon^128^. These differential characteristics are systematically compared in Table 3.

**Table 3:** Differential characteristics of PLM between patients with PD and those with idiopathic RLS

Feature	PLM in idiopathic RLS	PLM in PD
Comorbid RBD	Uncommon (17.2–17.9%)^129^	Common (31.6%), particularly in patients with a PLMI ≥ 15 (42.3%)^17^
PLMI during REM sleep	Typically low^130^	Elevated^15^
Asymmetric PLMS distribution and night-to-night variability	More prevalent^12^	Less prevalent^12^
Clinical presentation	1. Inter-limb movement intervals exhibit a characteristic bimodal distribution^131^ 2. Accompanied by a subjective urge to move and sensory discomfort^132^	1. PLMS are associated with executive dysfunction, with higher PLMI correlating with greater executive impairment^27^ 2. Frequently asymptomatic, detected only through objective monitoring^29^
Pathophysiology	Central nervous system iron deficiency and/or peripheral iron metabolism abnormalities^133^	Degeneration of nigrostriatal dopaminergic neurons^22,134^
Dopaminergic treatment responsiveness	Initially effective, but long-term use is associated with a significant risk of augmentation^135^	Effective, but therapeutic response is influenced by the wearing-off phenomenon^128^

*PD* Parkinson’s disease; *PLM* periodic limb movement; *PLMI*periodic limb movement index; *PLMS* periodic limb movements during sleep; *RBD* rapid eye movement sleep behavior disorder; *REM* rapid eye movement; *RLS* restless legs syndrome.

## Treatments

For PD patients with mild PLM, lifestyle interventions are considered first-line therapy. Maintaining a consistent daily schedule, creating an optimal sleep environment, avoiding caffeine, tea and alcohol before bedtime, and practicing relaxation techniques such as deep breathing, meditation or yoga are effective for managing PLM^136^. Furthermore, physical exercise has been demonstrated to significantly reduce PLMI values, achieving an effect comparable to levodopa^137^. However, it is important to note that engaging in physical activity within two hours before bedtime may increase the risk of PLM occurrence^138^. Thus, PD patients with PLM who exercise should avoid this period. When PLM is complicated by insomnia or insomnia-like symptoms, cognitive behavioral therapy for insomnia (CBT-I) can be employed to prevent exacerbation of PLM severity, an effect equivalent to pharmacological treatment with clonazepam^139,140^. In dietary management, maintaining a balanced diet and ensuring adequate intake of iron and magnesium can reduce the frequency and severity of PLM symptoms^42,141^.

While non-pharmacological therapies can alleviate PLM symptoms, dopamine agonists and levodopa remain the first-line pharmacological treatment^142^.

A recent study found that the rotigotine transdermal patch alleviated symptoms in patients with PLMD and improved sleep quality^109^. Notably, limb movements significantly decreased with an initial dose of 2.25 mg/day, and depressive symptoms unresponsive to conventional antidepressants disappeared after ten weeks. However, nightmares emerged when the dose was increased to 3.375 mg/day. Furthermore, depressive symptoms and limb movements reoccurred when the dose was decreased to 2.5 mg/day. As an example case, a newly diagnosed PD patient with severe PLMD (PLMI of 146.4/h) reportedly received treatment with the rotigotine transdermal patch, starting at 2 mg/day and titrated up to a maximum dose of 8 mg/day. After three months of consistent therapy, marked improvement in the PD symptoms and subjective sleep quality was observed, alongside a significant reduction in PLMS with a decrease in PLMI to 4.4 per hour^143^. A systematic meta-analysis demonstrated that rotigotine significantly reduced both the PLMI and PLM with arousal index compared to placebo groups. However, it was associated with a significantly higher withdrawal rate. Adverse events occurred in 60–73.9% of rotigotine-treated patients, with nausea being the most common, followed by headache, nasopharyngitis, application site reaction, somnolence, and dizziness^144^.

Similarly, a comparative study involving 12 PD patients demonstrated that transdermal administration of apomorphine achieved consistent drug delivery over 12 h, resulting in a 15% reduction in PLMI and alleviation of other PD symptoms including akinesia, rigidity, and sleep disturbances following a single night of therapy^145^. Other dopamine agonists or levodopa related drugs including ropinirole^146^, levodopa/Carbidopa/Entacapone fixed-dose combination^147^, and pramipexole^148^ have also been shown to alleviate PLM symptoms. Given their robust efficacy profile, dopamine agonists, particularly transdermal rotigotine, are considered a first-line therapeutic option for PD patients with comorbid PLMD.

The relationship between levodopa therapy and PLM in PD is complex. While dopaminergic treatment generally improves PLM, some studies have reported a higher prevalence of PLM in patients receiving lower levodopa equivalent doses or during the early stages of treatment initiation^28,149^. However, rather than being a direct side effect of levodopa administration, the appearance or exacerbation of PLM is increasingly recognized as a manifestation of the “wearing-off” phenomenon. A study reported a dose-related effect of dopaminergic medication in four PD patients with PLM. These individuals exhibited periodic, stereotyped lower extremity movements during the levodopa plasma trough, with symptom amelioration following administration of increased levodopa doses^128^, classifying this as a wearing-off phenomenon. Furthermore, Puligheddu et al. demonstrated that optimal levodopa treatment significantly reduced the PLMI below baseline levels, particularly before sleep onset^30^. Thus, PLM in treated PD patients may indicate insufficient nocturnal dopaminergic coverage rather than a toxic effect of the medication. Therefore, when PD patients present with PLM symptoms, particularly during the daytime, the preferred therapeutic strategy should prioritize dopaminergic medications to maintain therapeutic efficacy. However, it is essential to differentiate these movements from diphasic dyskinesia associated with levodopa use.

Compared to dopaminergic agonists for PLM, medications targeting alpha-2-delta ligands display comparable therapeutic efficacy. A clinical trial found that patients treated with gabapentin (initiated at 300 mg and titrated until symptom improvement) exhibited significant reductions in PLMS, PLMI, and PLMS with arousal index. In contrast, the ropinirole group showed reductions only in PLMS and PLMI. Furthermore, ropinirole treatment significantly altered sleep architecture, resulting in poorer sleep quality characterized by increased light sleep and reduced deep sleep, REM sleep, and total sleep time. Conversely, gabapentin treatment resulted in no significant changes in sleep structure and was notably associated with significant reductions in anxiety and depression scores^150^. Although gabapentin improved sleep quality and pain scores compared to placebo and reduced the prevalence of PLMI > 20 from 40.9% to 13.6%, it is notable that 48% of gabapentin-treated patients exhibited adverse effects including malaise, abdominal pain, somnolence, headache, and dyspepsia, a rate significantly higher than the 20.8% observed with placebo^151^. In addition, pregabalin, another alpha-2-delta ligand, has shown to ameliorate PLM symptoms. A double-blind, placebo-controlled trial demonstrated significant reductions in PLMI, PLM with arousal index, PLMW index, alongside improvements in sleep architecture. The recommended dose of 300 mg/day effectively alleviated PLM symptoms with mild tolerability issues, however, severe adverse events such as unsteadiness and daytime sleepiness emerged at a higher dose of 450 mg/day^152^.

Although alpha-2-delta ligands demonstrate efficacy in general PLM populations, clinical trials specifically investigating PLM in PD patients are currently limited. Therefore, efficacy evidence for the PD population is largely extrapolated, and these agents should be prescribed with caution given potential adverse effects such as somnolence and unsteadiness. Considering their impact on sleep architecture and anxiety, alpha-2-delta ligands serve as a valuable alternative first-line treatment, particularly for PLM in PD patients with comorbid insomnia, chronic pain, or anxiety, provided that fall risks are carefully managed.

Opioids are considered a second-line therapy for intractable PLM, showing efficacy in reducing PLMI and improving sleep architecture. However, it is necessary to note that current evidence is primarily derived from studies on idiopathic RLS or the general population, rather than specifically from patients with PD. Walters et al. reported that in patients with severe idiopathic RLS, switching to opioid monotherapy (propoxyphene for mild cases; oxycodone, codeine, or dihydrocodeine for moderate cases; and methadone for severe cases) resulted in sustained symptom relief and improved sleep quality over long-term follow-up^153–155^.

Despite these potential benefits, the use of opioids in PD patients should generally be discouraged or approached with extreme caution. Currently, there is a lack of clinical trial data regarding the safety and efficacy of opioids specifically for PLM in patients with PD. Furthermore, opioids carry significant risks of adverse effects, including addiction, sedation, constipation, and hallucinations, and may exacerbate respiratory depression^156^. In light of the potential risks regarding respiratory depression and gastrointestinal side effects, opioids should be reserved strictly as a last-resort therapy for refractory cases where all other standard treatments have failed, requiring close clinical monitoring^153^.

Currently, there are no available data or clinical trials specifically investigating iron supplementation for PLM in patients with PD. However, given the pathophysiological overlap between PLM/RLS and PD involving dopaminergic dysfunction and iron deficiency, therapeutic strategies are often extrapolated from guidelines for idiopathic RLS/PLMD. In the general population, oral iron supplementation, including ferrous sulfate (recommended dose: 3 mg/kg/day) and elemental iron (recommended dose: 65 mg, twice daily), is considered the first-line treatment. According to previous guidelines, iron supplementation is recommended for RLS patients with serum ferritin levels < 75 μg/L^62^. For children with PLMD, the goal of iron supplementation is to raise serum ferritin levels above 50 ng/ml^141,157,158^. When patients cannot tolerate oral iron or require urgent supplementation, intravenous iron therapy represents a valuable alternative^159^. The intravenous formulations used include iron sucrose (mean recommended dose: 3.6 mg/kg) and ferric carboxymaltose (recommended dose: 15 mg/kg, with a maximum dose of 750 mg)^159,160^. While these iron supplements have demonstrated improvements in PLM symptoms and reductions in PLMI in non-PD populations, large-scale clinical trials are necessary to confirm their efficacy and safety specifically in PD patients with PLM. Given this extrapolation, clinical trials are recommended and clinical monitoring is advised when initiating iron therapy in this population. Clinically, evaluating iron status is reasonable and optimizing iron levels in PD patients with serum ferritin below the normal reference range may be beneficial prior to escalating to other pharmacological therapies.

Selegiline, a type-B monoamine oxidase inhibitor used to treat PD, may also be beneficial for improving NMS^161^. A retrospective observational study of 31 PLMS patients treated with selegiline reported a significant reduction in the PLMI from 35.6 to 14.5 with mild side effects^162^. Furthermore, melatonin, another therapeutic agent for PD, has been shown to significantly improve PLM symptoms and reduce PLMI in seven out of the nine PLMD patients without RLS^98^. Additionally, antiseizure medications (valproate^163^), and benzodiazepines^164^ were reported to alleviate PLM symptoms, reducing both the PLMI and PLM-arousal index. Collectively, these agents might serve as viable adjunctive options, particularly for patients who require simultaneous management of motor symptoms or sleep rhythm disturbances, although their specific efficacy in the PD-PLM population warrants further validation through larger controlled trials.

When conventional pharmacological therapy fails to adequately control PLM symptoms, surgical approaches such as deep brain stimulation (DBS) and stellate ganglion block (SGB) may serve as alternative therapeutic strategies^90,165,166^. In a reported case of complicated PLMD unresponsive to multiple medications, SGB resulted in marked improvement of limb movements, presumably through blockade of the stellate ganglion, reduction of sympathetic activity, and subsequent attenuation of PLM^90^. Similarly, in a patient with PD and comorbid PLM refractory to medical therapy, globus pallidus internus DBS led to resolution of PLM symptoms and enabled substantial reduction of PD medication at one-month follow-up. At two years postoperatively, PD symptoms remained significantly improved and PLM symptoms continued to be absent^166^. One functional magnetic resonance imaging (fMRI) study demonstrated that spontaneous PLM episodes are associated with activation in the red nuclei and brainstem, without cortical involvement^104^. However, voluntary imitation of this movement triggers activation in the globus pallidus and motor cortex rather than the brainstem. These findings indicate that functional dysregulation of the basal ganglia-thalamocortical circuit may play a significant role in the pathogenesis of PLM; nevertheless, further clinical trials are required to substantiate this conclusion.

**Fig. 2:**
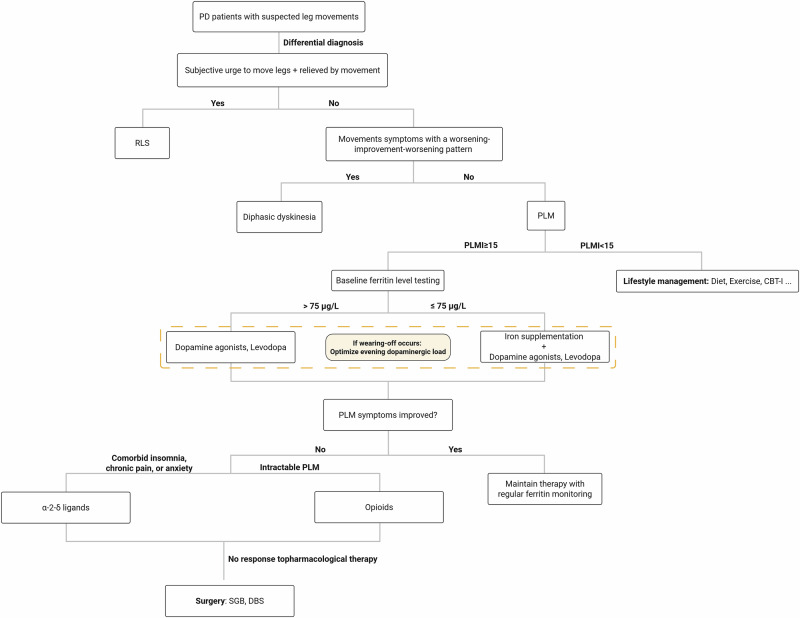
A stepwise clinical decision tree for the diagnosis and management of PLM in PD. The flowchart begins with the differential diagnosis, distinguishing PLM from RLS and diphasic dyskinesia, which may exhibit overlapping clinical presentations. Following a confirmed PLM diagnosis, treatment is stratified based on the PLMI. For patients with a PLMI < 15, lifestyle management (e.g., diet, exercise, and CBT-I) is recommended, whereas those with a PLMI ≥ 15 undergo baseline serum ferritin level testing. For patients with ferritin levels ≤ 75 μg/L, iron supplementation combined with dopamine agonists or levodopa is recommended as first-line therapy; for those with ferritin levels > 75 μg/L, dopamine agonists or levodopa are initiated directly. Notably, the evening dopaminergic load should be optimized if the wearing-off phenomenon occurs during treatment with dopamine agonists or levodopa. Responders are maintained on current therapy with regular ferritin monitoring. For non-responders with comorbid insomnia, chronic pain, or anxiety, α-2-δ ligands are recommended as second-line therapy. Opioids are reserved for intractable PLM, and surgical interventions, including SGB and DBS, are considered only for patients refractory to all pharmacological treatments. Abbreviations: CBT-I, cognitive behavioral therapy for insomnia; DBS, deep brain stimulation; PD, Parkinson’s disease; PLM, periodic limb movement; PLMI, periodic limb movement index; RLS, restless legs syndrome; SGB, stellate ganglion block.

To assist clinicians in the systematic management of PLM in PD patients, a clinical decision algorithm is proposed (Fig. 2). The algorithm initiates with a differential diagnosis to exclude RLS and diphasic dyskinesia, followed by treatment stratification based on PLMI values. For patients with a PLMI ≥ 15, baseline serum ferritin assessment guides first-line therapy selection: iron supplementation combined with dopaminergic agents is recommended for those with ferritin ≤75 μg/L, whereas dopaminergic agents alone are initiated for those above this threshold. Subsequent treatment escalation proceeds through α-2-δ ligands and opioids, with surgical intervention reserved for refractory cases.

## Future directions

Despite the growing body of research on the association between PLM and PD, several critical and testable research gaps persist. Addressing these issues would deepen the mechanistic understanding of PLM-PD comorbidity, improve diagnostic accuracy, optimize clinical management, and provide a framework for future clinical trial design.

First, large-scale, multicenter, prospective cohort studies employing a standardized PLMI threshold (≥15 events/hour) and controlling for confounders such as dopaminergic medication use and sleep-related comorbidities are needed to establish the true prevalence of PLM across PD stages and among diverse racial and ethnic populations. Furthermore, it remains unclear whether an elevated PLMI in RBD or prodromal PD cohorts can serve as an independent predictive biomarker for phenoconversion to clinically established PD, and if so, what the optimal diagnostic PLMI cutoff should be. Additional investigations are also required to confirm whether age and sex act as independent risk factors for PLM within the PD population.

Mechanistically, the pattern of cerebral iron redistribution in patients with PLM–PD comorbidity has not been fully characterized, and it remains to be established whether current or emerging neuroimaging modalities can provide reliable quantitative and qualitative assessments of such redistribution. It is also unknown whether the PLMI correlates with the severity of pathological α-synuclein aggregation within brainstem nuclei (e.g., the locus coeruleus, raphe nuclei, and pedunculopontine nucleus) or the degree of neuronal loss in the A11 dopaminergic cell group.

Regarding therapeutic strategies, the efficacy of oral or intravenous iron supplementation in reducing the PLMI among PD patients with documented low serum ferritin (<75 μg/L) warrants immediate investigation. Despite being a readily testable intervention, clinical trial data specifically targeting the PD population remain lacking. Furthermore, the optimal dopaminergic regimen, including dosage, formulation, and timing of administration, for managing nocturnal PLM in PD while minimizing wearing-off phenomena and symptom augmentation has not been defined. Head-to-head randomized controlled trials are essential to compare the efficacy and safety profiles of dopamine agonists versus levodopa formulations in this specific clinical context.

## Conclusion

PLM is a common sleep disorder in individuals with PD, significantly impairing both daytime function and sleep quality. This review summarizes the prevalence, risk factors, clinical manifestations, pathophysiology, diagnosis, and treatment of PLM in PD.

Multiple factors, including gender, age, inflammation, HP, and genetics may increase the prevalence of PLM in PD patients. PD patients with comorbid PLM exhibit more severe clinical manifestations, particularly increased sleep disturbances, cognitive dysfunction, and psychiatric symptoms. The underlying pathophysiology potentially involves iron deficiency, dopaminergic dysregulation, sympathetic nervous activity, dysfunction of circadian rhythms and brainstem nuclear impairment. PSG remains the gold standard for diagnosing PLM, while actigraphy and the PAM-RL device serve as alternative methods.

Although dopamine agonists and levodopa are principal pharmacological treatments for PLM, the efficacy of levodopa, specifically in PD patients with PLM, remains controversial and requires further clinical trials to establish its long-term effectiveness. However, several limitations of this review should be acknowledged. First, due to the scarcity of large-scale, PD-specific clinical trials, substantial portions of the pathophysiological mechanisms and therapeutic evidence were extrapolated from studies on RLS or the general population. Therefore, these mechanistic conclusions should be interpreted cautiously. Second, most included studies were observational or cross-sectional with relatively small sample sizes, limiting the ability to establish definitive causal relationships between PLM severity and PD progression.

Current research on PLM in PD is limited, and the efficacy of pharmacological treatments such as iron supplementation requires more clinical trials to be demonstrated in this population. Therefore, elucidating the pathophysiology of PLM is necessary for developing targeted therapeutic strategies for PD patients with PLM.

## Data Availability

No datasets were generated or analysed during the current study.

## Code availability

Code availability is not applicable to this article as no code was generated or analyzed during the current study.
